# The Use of Photodynamic Therapy for Head, Neck, and Brain Diseases

**DOI:** 10.3390/ijms241411867

**Published:** 2023-07-24

**Authors:** Wojciech Domka, Dorota Bartusik-Aebisher, Wiktoria Mytych, Klaudia Dynarowicz, David Aebisher

**Affiliations:** 1 Department of Otolaryngology, Medical College of The University of Rzeszów, 35-959 Rzeszów, Poland; w.domka@gazeta.pl; 2 Department of Biochemistry and General Chemistry, Medical College of the University of Rzeszów, 35-959 Rzeszów, Poland; dbartusikaebisher@ur.edu.pl; 3 Students English Division Science Club, Medical College of The University of Rzeszów, 35-959 Rzeszów, Poland; wiktoriamytych@gmail.com; 4 Center for Innovative Research in Medical and Natural Sciences, Medical College of The University of Rzeszów, 35-310 Rzeszów, Poland; kdynarowicz@ur.edu.pl; 5 Department of Photomedicine and Physical Chemistry, Medical College of the University of Rzeszów, 35-959 Rzeszów, Poland

**Keywords:** photodynamic therapy, head diseases, neck diseases, brain tumor, treatment

## Abstract

Head–neck cancers as a group have the 7th highest rate of incidence worldwide. The most often diagnosed disease of the head and neck is squamous cell carcinoma (90% of cases). Another specific group of tumors is brain tumors. These can be divided into primary tumors and secondary tumors associated with metastasis. Research shows that treating head and neck cancers continues to be problematic and challenging, and researchers are actively seeking new treatments that would improve survival rates and reduce side effects. Irradiation of tumor tissue with the optimal wavelength of light in photodynamic therapy (PDT) generates predominantly singlet oxygen in tissue-based photosensitizers (PSs) or reactive oxygen radicals in the case of vascular PSs leading to cellular apoptosis and necrosis. A very important feature of PDT is that cells cannot become immune to the effects of singlet oxygen or reactive oxygen radicals. However, photosensitizer (PS) transport is influenced by the specific structures of cancer tumors and the concentration of PS decreases in cells far from the vessel lumen. Therefore, PSs may not reach tumor interiors, which decreases therapy effectiveness. The use of drug carriers and 3rd generation PSs that contain biocompatible functional groups makes it possible to control transport. This review of the current literature on PDT was conducted through databases such as PubMed and Scopus. The types of publications considered included clinical studies and most of the articles included were published in English. Based on the publications collected, we conclude that researchers have demonstrated the potential of PDT as a therapeutic platform for head, neck, and brain diseases.

## 1. Introduction

Due to lifestyle changes that increase exposure to risk factors, the number of diseases in the general population, including cancer, is steadily increasing [1]. One group of cancers is head and neck cancers (HNCs). In Poland, HNCs are diagnosed in about 11,000 people per year, and over the past 10 years, the number of patients with HNC has increased by 25% [2]. Head–neck cancers as a group, have the 7th highest rate of incidence worldwide [3]. Head–neck cancers include cancers of the lip, mouth, pharynx, larynx, salivary glands, nasal sinuses, nasal cavity, auditory organs, and the brain. One of the most common cancers in this location is cancer of the larynx. According to published studies, the incidence of HNC is three to four times higher in men than in women and 90% of cancers of the head and neck region are squamous cell carcinomas (SCC) [4]. The etiology of HNC is said to be influenced by toxic external substances, such as cigarette smoking and alcohol consumption. Studies show that regular smoking in conjunction with alcohol consumption significantly increases the risk of these types of cancer. Other causes of HNC include air pollution, UV light exposure, viral infections, or mechanical irritation. The role of human papillomavirus HPV in the etiology of HNC has been discussed at length. Additionally, the increased incidence of HNC among young people may be associated with changes in sexual habits or reduced vaccination rates [5]. Genetic factors, environmental factors, lifestyle, poor nutrition, and a sedentary lifestyle have also been implicated as factors in the etiology of HNC [6]. The symptoms of HNC depend on the location of the tumor. In the case of the oral cavity or pharynx, there are difficulties swallowing and hoarseness. In the case of ear cancer, there may be hearing loss or balance disorders.

Cancer of the nasal cavity makes breathing difficult and impairs the sense of smell. The anatomical location of the tumor in the cervicothoracic area affects some of the most important functions of life, namely, eating, breathing, and speaking [7], and the impact on the patient’s appearance can compound the psychological burden. Another specific group of tumors is brain tumors, which are generally divided into primary and secondary tumors associated with metastasis. Primary tumors can originate from glial cells, meninges, choroid plexus, the pituitary gland, or blood vessels. Metastatic tumors are most often transferred from the lungs and breasts. The most common symptoms include headaches and seizures, which are associated with increased intracranial pressure [8]. Genetic factors and ionizing radiation have been implicated in the etiology of primary brain tumors. Non-malignant meningiomas or meningiomas and pituitary tumors account for 50% of primary brain tumors. One primary brain tumor that is associated with high malignancy and a poor prognosis is glioblastoma multiforme (GBM). GBM accounts for 15% of primary brain tumors. Statistics show that after a brain tumor diagnosis, only one in three people survive for at least 5 years [9]. Research shows that treating head and neck cancers continues to be problematic and challenging, and researchers are actively seeking new treatments that would improve survival rates and reduce side effects. Such challenges may be met by advancements in boron neutron capture therapy (BNCT) and photodynamic therapy (PDT). Boron neutron capture therapy is a minimally invasive therapy. Its main principle of operation is the process of neutron fission. This process releases alpha particles, which selectively damage cancer cells. Currently, there are several reports describing this method, however, new support systems are still being sought and developed. One of the main research objectives is to characterize the selective delivery of therapeutic concentrations of boron atoms to tumor tissues. According to the authors of one study, it is the liposomes that are the best developed and researched in terms of the use of oncological therapy. Overall, the development of targeted liposomal systems seems to be the best option as they allow for active targeting, which can improve tumor distribution, and have a high drug-loading capacity, which enables the incorporation of anticancer agents [10]. In a report by Burian et al., patients with glioblastoma were given boron neutron therapy, and tissue samples were analyzed by mass spectroscopy. Patients were treated in the epithermal neutron plant of a reactor. The maximum dose did not exceed 14.2 Gy-Eq. The study showed good tolerance by the patients of the method used [11]. A similar study was conducted by Yamamoto et al. Nine patients aged 18–70 years with glioblastoma of various grades were treated with non-surgical boron neutron capture therapy. Neutron irradiation was performed in a single fraction using a mixed thermal/epithermal beam. The project covered the years 1999–2002. The maximum dose of boron was in the range of 11.7–12.2 Gy in the brain and was associated with radiation necrosis. The authors report that radiation necrosis developed within the target tumor volume, which included 2 cm beyond the surgical margin or residual tumor. The clinical application of the developed method was determined to be safe and effective [12].

The origins of PDT date back to 1900. Oscar Raab made the accidental discovery that microorganisms that were incubated with certain dyes could be killed when exposed to light. It was also noted that oxygen was necessary to kill these cells, and thus the term “photodynamic action” was coined. Following these discoveries, the effectiveness of PDT as a cancer therapy began to be studied. PDT is increasingly being used in modern medicine and represents a modality with high potential due to its highly selective mode of action that is focused primarily on the affected tissue. This precise action allows healthy cells to be spared [13,14]. Photosensitizers (PSs) have no dark toxicity and only initiate photodynamic action when exposed to light [15]. PDT is an emerging treatment modality that holds promise as a progressive method of selective therapy for head and neck cancers and has been primarily used in dermatology and ophthalmology. PDT requires the presence of three components: a photosensitizer (PS), oxygen, and light of appropriate wavelength for PS absorption and excitation. PSs are of two main types: tissue-based, which accumulate in organelles of cancer cells, and vascular, which circulate in the bloodstream and destroy tumor vasculature upon exposure to light. Cellular hypoxia is a limiting factor in the effectiveness of PDT as photodynamic action requires endogenous dissolved oxygen. Cellular hypoxia can affect the transport of drugs through various pathways, e.g., low oxygen can affect cellular function and alter drug action or metabolism and hypoxia could potentiate or mitigate drug-induced toxicity. Therefore, cellular oxygen availability could be a cofactor to manipulate in the treatment of hypoxia-induced pathology [16,17].

Minimally invasive PDT requires the presence of PS, light, and oxygen. It involves the oxidation of biomolecules by singlet oxygen or other reactive oxygen species (ROS), such as the hydroxy radical, in a selective manner in the presence of dissolved molecular oxygen in the cells. The PS may be selectively retained in tissue lesions or present in the tumor vasculature prior to exposure to light leading to the destruction of tumor tissues or tumor vasculature with an accompanying immune response [13,18,19,20]. The mechanism of PDT is dependent on the concentration of PS, oxygen, and light dose. It is generally accepted that low-dose PDT induces apoptotic cell death while high-dose PDT mostly causes necrotic cell death. Photodynamic therapy is referred to as doubly selective, first, by its ability to limit applied radiation to a specific volume of tissue, and second, by the lack of PS dark toxicity. For photoactivation, the wavelength of light is matched to the absorption of the PS so that the photons are absorbed and the PS can either transfer the energy gained to ground-state oxygen or undergo electron transfer processes [21]. Although the PDT method has many limitations, it is still more selective than traditional surgical therapies or chemotherapy. Most chemotherapeutics are hydrophobic and require solvents to formulate the dosage form, which contributes to the severe toxicity and lack of selectivity of anticancer drugs Chemotherapy also suffers some limitations related to aqueous solubility [22]. One of the biggest limitations in nanoparticle-aided drug delivery is clearance by the reticuloendothelial system (RES) through opsonization and it is implicit here that the size influences clearance as well as distribution [23]. PDT is less invasive than surgery. It usually takes only a short time and is most often done as an outpatient procedure. It can be targeted very precisely. Unlike radiation, PDT can be repeated many times at the same site if needed.

PDT is an alternative to major surgery and is often used as an adjunct to chemotherapy and radiotherapy [24,25]. The purpose of the current study was to evaluate the effects of PDT in the treatment of diseases including head and neck cancers, as well as brain tumors or other diseases that are in a precancerous stage. Using the desired physicochemical reactions ultimately leads to the apoptosis of tumor tissue. Numerous studies are currently being conducted to refine PDT and evaluate its effectiveness. Conducted by chemists, biologists, physicists, doctors, and engineers, they make the investigation of PDT a highly multidisciplinary field.

### General Principles of Photodynamic Therapy

The treatment of cancerous tissue by PDT involves the selective oxidation of biological material by singlet oxygen or reactive oxygen-derived radicals. Singlet oxygen is not only toxic to cells and impairs signaling events but is also capable of eliciting a cellular stress response [26].

PDT is a light-oxygen-controlled process that has no toxicity in the absence of oxygen or light, and tissue oxygenation levels must be sufficient to sustain the formation of ROS. The PS is non-toxic as long as it is not irradiated, and is introduced into the body either intravenously or in topical solutions for the treatment of skin diseases. The effect of PDT depends on both the concentration of PS and the light dose delivered. Tissue is photodamaged if the concentrations of PS oxygen and light are high enough. Tissue necrosis occurs if the threshold dose of PDT is exceeded. All death receptors belong to the tumor necrosis factor receptor (TNFR). The crucial factors in determining the type of cell death, e.g., apoptosis or necrosis following PDT are: the cell type, the presence of an intact set of apoptosis machinery, the subcellular localization of the PS, the light dose applied to activate it locally, and the oxygen partial pressure [27,28].

The characteristics of selected PSs are given in Table 1.

**Figure 1:**
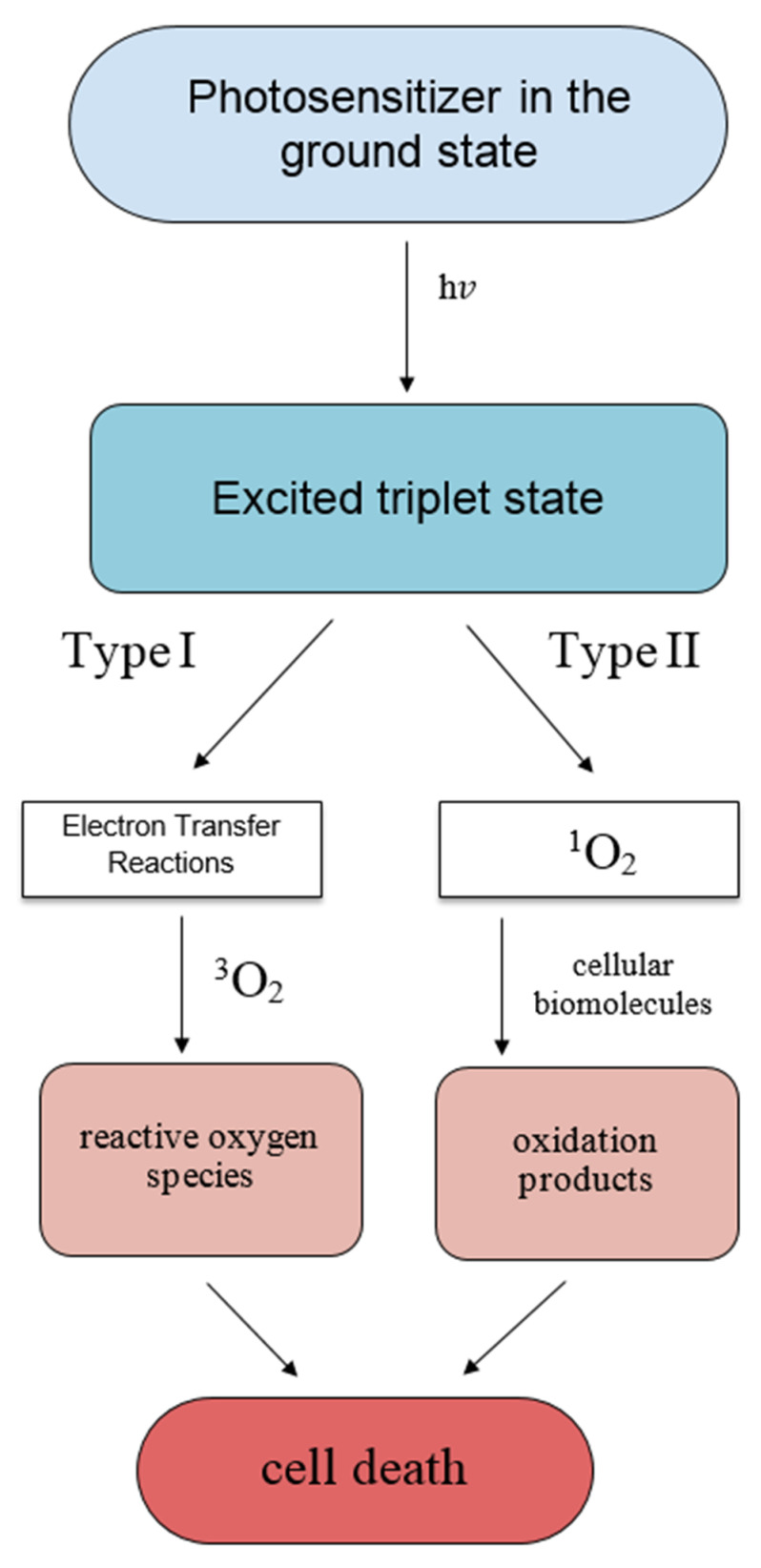
Type I and Type II processes. Type I: The photosensitizer in the triplet state undergoes electron transfer processes leading to formation of cytotoxic hydroxy radical, superoxide, and hydrogen peroxide. Type II: photosensitizer in the triplet state transfers energy directly to ground-state triplet oxygen to generate singlet oxygen.

PSs decompose through bleaching, although irradiation times are usually brief (minutes) so the therapy does not lose its effectiveness and continues tumor destruction [29]. It is important to start the irradiation stage when the PS has either been absorbed into cells, which may take up to several days in the case of tissue-based PSs, or within minutes of injection for vascular PSs [30]. Thus, the parameters of PDT include PS degradation, differential tissue uptake, light dose, oxygen concentration, and threshold effects [31]. A PS operates by light absorption, which results in the formation of an excited singlet state that rapidly intersystem crosses to the excited triplet state [32]. The triplet state is unstable and loses energy through internal conversion, phosphorescence, or by transfer through collision with molecular oxygen to form singlet oxygen [33]. This energy transfer pathway is referred to as a Type II photochemical process leading to the generation of singlet oxygen. ROS can also be formed through electron transfer reactions in pathways referred to as a Type I photochemical process. This Type I mechanism generates reactive oxygen radicals such as the hydroxy radical that are also cytotoxic and is the accepted mode of action by vascular PSs [34]. The type of mechanism also depends on the concentration of oxygen in the immediate environment. With PDT, cancer cell death occurs through necrosis or apoptosis. Necrosis tends to occur when there is a high concentration of PS and the injection time is short [13]. Apoptosis tends to occur in the presence of low concentrations of PS with long incubation times. Necrosis is irreversible damage to a cell and its functions, while apoptosis is the planned death of a cell. Both mechanisms ultimately lead to the death of cancer cells. Molecular oxygen (which has two unpaired electrons) exists as a triplet ground state and the more reactive singlet oxygen formed in the aforementioned Type II process through energy transfer is the mode of action of most clinically approved PSs [35,36]. Type I and Type II is presented in Figure 1.

Both Type I and II photo processes may occur at the same time, and their ratio depends on the type of PS used and the local concentration of oxygen [24]. PSs excited by light can be inactivated in three primary ways: the absorbed energy can be converted to heat, fluorescence, or phosphorescence [36,37].

A PS is a dye that is essential for PDT therapy. Its action is to selectively accumulate in the affected tissue and sensitize it to light [38]. Thus, when exposed to radiation of a given wavelength, singlet oxygen is generated, which destroys the cells in which the PS has accumulated. Desirable characteristics that a PS should exhibit are selectivity, high quantum yield of ROS, low dark toxicity, non-mutagenicity, minimal patient light sensitivity post-PDT, a suitable method of injection, rapid clearance, and relative hydrophobicity for ease of transport in the body. Also important for tissue-based PSs are preferential uptake by target cells and localization in the mitochondria or endoplasmic reticulum [39]. Before incubation, the PS is often diluted to achieve the desired final concentrations. PSs have multiple light absorption peaks, including several Q-bands and a Soret band, which may range from 400–800 nm depending on the PS. Absorption in the near-infrared is preferred for PDT due to the deeper penetration of near-infrared light radiation into tissue [40]. The characteristic feature of a given PS is the concentration c (measured with a spectrophotometer) and the extinction coefficient ε, which are related by A = ε c l, where A is the absorbance determined spectrophotometrically. PSs with longer wavelength absorptions and higher molar absorption coefficients are more efficient. Absorption of single photons with wavelengths longer than 800 nm does not produce enough energy to excite oxygen to the singlet state. Exposure to the appropriate wavelength of light causes the PS to transition from the low-energy ground state to the excited singlet state [19,41,42]. PSs can be further divided into three generations [43]. First-generation PSs are porphyrin-based hematoporphyrin derivatives (HpD) such as Photofrin^®^, and their main disadvantage is poor light absorption in the red spectral region. Photofrin^®^ (Porfimer sodium; Axcan Pharma, Inc., Mont-Saint-Hilaire, QC, Canada) was the first approved PDT agent for the treatment of obstructive esophageal cancer in 1995 [44].

Second-generation PSs are mostly modified porphyrin- or chlorin-based compounds with longer absorption wavelengths and less photosensitization of the skin after treatment. Third-generation PSs are still in the development stage and are second-generation PSs modified with various biocompatible molecules (i.e., sugars, PEG, proteins) to increase tumor specificity [36]. The use of natural compounds seems contradictory because plants evolved to grow in sunlight so they cannot contain highly active PSs; however, there are several isolated natural products, e.g., hypericin, riboflavin, and curcumin, that are currently being investigated. A number of second-generation PSs have been developed over recent decades, including Motexafin lutetium (Lutrin^®^ and Lutex^®^; Pharmacyclics Inc., Sunnyvale, CA, USA), Temoporfin (Foscan^®^; Biolitec AG, Jena, Germany), Palladium bacteriopheophorbide (Tookad^®^; Negma-Lerads, Magny-Les-Hameaux, France), purpurins (Purlytin^®^, Pfizer, New York, NY, USA), Verteporfin (Visudyne^®^; Novartis, Basel, Switzerland), and protoporphyrin IX (PPIX) precursors (Hexvix^®^, Photo cure, ASA, Oslo, Norway, Metvix^®^/Metvixia^®^, Photo cure, ASA, Oslo, Norway, and Levulan^®^, Dusa Pharmaceuticals, Wilmington, MA, USA). Foscan^®^ (Biolitec AG, Jena, Germany) is a second-generation photosensitizing agent extensively used for the treatment of head and neck cancer [45].

Nanotechnology allows the delivery of nanoparticles, fullerene-based PSs, and titanium oxide photocatalysts, and the use of upconverting nanoparticles to increase light penetration into the tissue [25,46]. Second-generation PSs did not show enough tumor selectivity; thus, many studies focus on third-generation PSs that show higher tumor specificity with long-wavelength light activation. This can be achieved by conjugation or encapsulation of existing PSs in carriers that can be delivered to the target tissue, and novel third-generation PS conjugated with antibodies are being developed for specific tumor tissue targets [47,48,49,50,51,52]. Some examples of second-generation PSs are presented in Table 2.

## 2. Methodology

**Figure 2:**
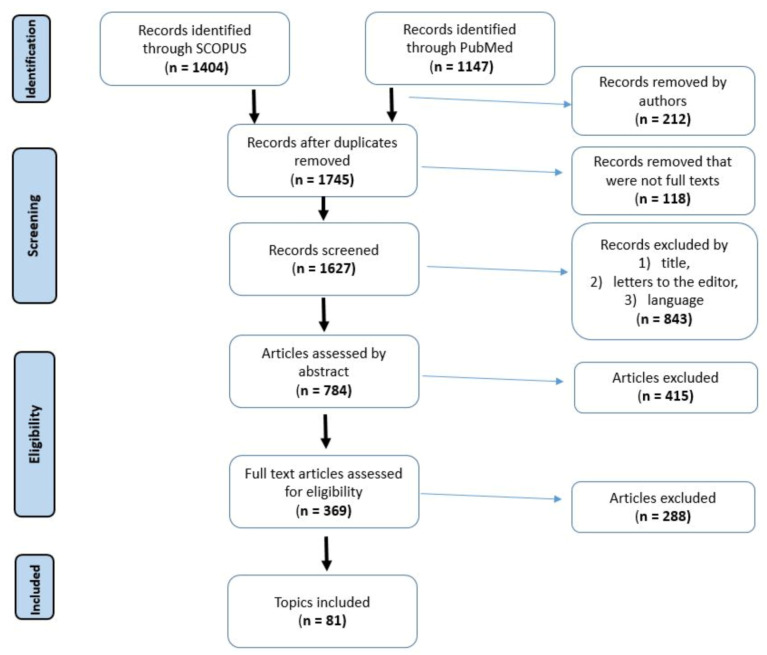
PRISMA flow diagram of included studies.

A search focused on the efficacy of PDT in the treatment of head, neck, and brain diseases was conducted on Pubmed and Scopus from inception to June 2023. This review was conducted based on the Preferred Reporting Items for Systematic Reviews and Meta-Analyses (PRISMA) guidelines [53]. The search term included the phrases “photodynamic therapy in brain diseases” and “photodynamic therapy in neck diseases”. The authors of this review worked on the basis of an agreed scheme, selecting articles based on their title, language, abstract, and access. Duplicate records were removed. PRISMA flow diagram is presented in Figure 2.

## 3. A Review of the Literature

For the analysis of relevant scientific publications, the authors of the works and their results are indicated. All selected papers include in vivo and/or in vitro studies. Most of the studies reviewed were concerned with the impact of the application of photodynamic therapy for various cancers of the head, neck, and brain.

### 3.1. Brain Diseases

Zilidis et al. presented a study that included six patients with metastatic brain melanoma. The mean age of the patients was 52.8 years. All patients had dexamethasone 4 mg/kg Qid and ranitidine 150 mg BD implemented a few days before surgery. Forty-eight hours prior to surgery, patients received 2 mg/kg body weight of Photofrin^®^ (Axcan Pharma, Mont-Saint-Hilaire, QC, Canada) intravenously, and 3 h before surgery they received 20 mg/kg body weight of 5-aminolevulinic acid (ALA, medac, Hamburg, Germany). The tumor was removed using intraoperative navigation, fluorescence-guided resection, and repeated photodynamic therapy. Across 5 days, the total dose of light was 500 J/cm^2^. The median total light dose was 394.91 J per day. The authors note that two postoperative complications occurred during the study: deep vein thrombosis and balloon catheter collapse. However, they stress that none of the patients experienced surgical death, infection, or neurological loss. Fifty percent of the subjects died of malignant melanoma located elsewhere, and Fifty percent died of unrelated causes. None of the patients had brain disease at the time of death. The median survival was 50 weeks, and the mean survival was 34.8 weeks. In this work, PDT was found to be an effective treatment option for metastatic brain melanoma [54].

**Figure 3:**
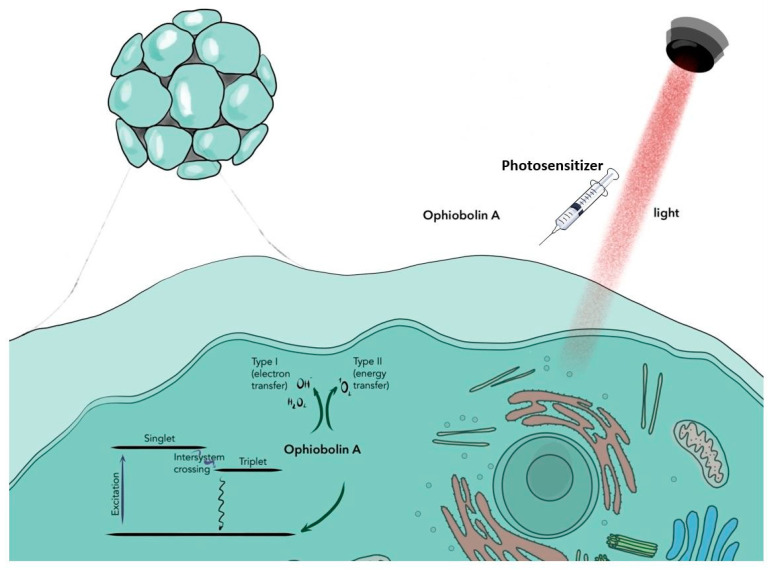
Therapeutic mechanism of PDT with ophiobolin A in glioblastoma.

Stylli et al. included 358 patients diagnosed with GBM in their study. The study sample consisted of 136 patients who underwent surgical treatment for GBM and anaplastic astrocytoma (AA). The median age was 40 years. All deaths were due to recurrent brain tumors. In 78 patients, it was glioblastoma multiforme, and in the remaining 58, it was an anaplastic stellate tumor. Patients with initially treated tumors received standard postoperative radiation therapy, and patients with tumor recurrence were previously treated with radiation therapy. Chemotherapy was used in 29% of patients. Patients were given intravenous 5 mg/kg HpD 24 h before surgery for 30 min. Initially, they used a dose of 70 J/cm^2^, which was later increased to 240 J/cm^2^. After tumor resection, 0.5% aqueous lipid suspension Intralipid (Baxter Healthcare, Old Toongabbie, NSW, Australia) was used to fill the resulting cavity. In patients with primary tumors, the median survival from initial diagnosis was 76.5 months for AA and 14.3 months for GBM. Seventy-three percent of AA patients survived more than 36 months. Twenty-eight percent of patients with GBM survived more than 24 months, and twenty-five percent survived more than 36 months. For patients with recurrent cancer, the median survival after repeat surgery was 66.6 months for AA and 14.9 months for GBM. Sixty-one percent of patients with recurrent AA survived more than 24 months and fifty-seven percent more than 36 months. Forty-one percent of patients with recurrent GBM survived more than 24 months and thirty-seven percent more than 36 months. The authors note that there were no immediate serious complications after PDT in their study. However, one patient died of acute myocardial infarction 15 days after surgery. Half-paralysis in one patient and increasing lethargy and hemiparesis in three patients were also reported. The studies presented here show promising results for the use of PDT on increasing median patient survival in both primary and recurrent glioma patients [55]. Recent studies show that ophiobolin A is a new effective anticancer agent in the treatment of GBM. Ophiobolin A is a secondary metabolite of fungal origin that has enhanced activity against GBM cells known to be resistant to apoptosis. Studies confirm that the applied ophiobolin A inhibits tumor growth in target cancer cells. Because glioblastoma cells are extremely resistant to standard therapeutic methods, there is a great need for more effective therapeutic agents and techniques. PDT supported by ophiobolin A is a promising treatment, although not yet fully developed [56,57]. Figure 3 shows the therapeutic mechanism of PDT with ophiobolin A in glioblastoma.

Akimoto et al. included 14 adult patients with malignant gliomas (6 patients with primary tumors, 8 patients with tumor recurrence) with infiltration into the eloquent areas of the brain in their study. PDT was used as an additional intraoperative treatment after craniotomy and tumor resection. A total of 40 mg/kg of intravenous talaporfin sodium was used 24 h before surgery. The tumor cavity after resection was irradiated with a 664 nm diode laser for 180 s at a power density of 150 mW/cm^2^. Six patients with primary tumors showed better treatment efficacy, with about an 80% success rate. In these patients, the median tumor progression-free survival time was 23 months. The median survival time in the three patients who died was 26 months, and the remaining three patients survived for 3 years. In patients with tumor recurrence, efficacy was low (only 25%). The glioma recurrence occurred 3 months after PDT, and the survival time was 9 months. The authors confirm the safety and efficacy of PDT with talaporfin sodium, especially in patients with primary gliomas [58].

Marks et al. examined the effectiveness of PDT on 12 patients with recurrent pituitary adenoma. All patients were given Photofrin^®^ systemically and after 24–48 h underwent irradiation of the tumor cavity with 630 mm laser light in an intraoperative setting. The decrease in tumor volume over time compared to the preoperative size was 122, 87, 66, 60, and 46%. Most patients reported improvements in vision and endocrine function. In three patients, there was a complete restoration of the visual field. The authors report that the only adverse effects were minor skin reactions. One patient suffered hemiparesis but recovered completely. The authors believe that this is not related to the treatment they used. The authors emphasize the effectiveness and good tolerability of PDT [59].

In a study by Igbaseimokumo et al., the goal was to quantify the preferential uptake of Photofrin^®^ by pituitary adenoma tissue for intraoperative PDT. Eight patients with recurrent pituitary adenoma were studied. All were given 2 mg/kg of Photofrin^®^ intravenously 48 h before surgery. During the procedure, tissue samples were taken from the pituitary adenoma, muscle, fat, skin, and plasma. These samples were evaluated by a fluorometric assay. The average levels obtained for Photofrin^®^ were 6.87 ng/mg for pituitary adenoma, 2.24 ng/mg for skeletal muscle, and 2.54 ng/mg for fat. Skin samples showed an uptake of 2.19 ng/mg; however, these were taken from only four patients. The authors showed that Photofrin^®^ is suitable for intraoperative PDT and is preferentially retained in pituitary adenoma tissue. They also report that the plasma concentration of 7.65 ng/mg was comparable to that of the drug in the pituitary adenoma. In vivo, Photofrin^®^ has a threefold higher uptake than in normal tissues like muscle and fat [60].

Stepp et al. conducted a study of fluorescence-guided resection and PDT in patients with malignant gliomas. They used 20 mg/kg of 5-ALA orally, obtaining results of average PPIX fluorescence with live tumors more than 100 times that found in the normal cerebral cortex. The contrast-enhanced tumor was completely removed in 65% of patients in the 5-ALA group. The authors showed that 5-ALA-PDT can have a very safe and effective therapeutic effect in treating primary or secondary gliomas or even for those classified as inoperable [61].

Muragaki et al. included 27 patients with suspected malignant parenchymal brain tumors in their study. Patients were given 40 mg/kg of talaporfin sodium intravenously. Photodynamic therapy was applied to the resection site the day after tumor removal. Twenty-two patients with a histopathologically confirmed diagnosis of primary malignant parenchymal brain tumor were included in the study. One of the twenty-two patients died 3.4 months after resection and PDT. The 12-month overall survival (OS) rate was 95.5%. The 6-month progression-free survival time (PFS) and local PFS were 91%. Patients were followed up for 38.6 months. Median OS was 27.9 months, median PFS was 20 months and local PFS was 22.5 months. Thirteen of the diagnosed tumors turned out to be GBM in which the 12-month and 6-month local PFS rates were 100%. A study using intraoperative PDT based on the administration of talaporfin sodium showed a positive effect on the treatment of primary malignant parenchymal brain tumors. The therapy increases tumor control and prolonged the lives of patients [62].

Muller and Wilson included 20 patients with newly diagnosed malignant supratentorial glioma in their study. The average age was 56 years (female to male ratio 1:1). Eleven patients were diagnosed with GBM and nine with malignant glioma (MA). All patients were given intravenous porphyrin PS 12–36 h before surgery, followed by tumor resection and cavity irradiation. Median survival for all subjects was 44 weeks. Patients with GBM had a median survival of 37 weeks, while those with MA had a median survival of 48 weeks. No complications occurred in 85% of the participants. There were two deaths and one patient had a persistent neurological deficit. Despite the poor prognosis of patients diagnosed with GBM and MA, the authors emphasize the safety of PDT therapy, which, when used appropriately, did prolong survival in some patients [63].

### 3.2. Head, Neck, and Oral Diseases

Biel et al. report that photodynamic therapy is an effective treatment for early head and neck cancers. They tested PDT on 87 patients with cancers of the larynx, oral cavity, pharynx, and skin. Patients were followed up for 66 months. Patients with T1 cancer and carcinoma in situ received a complete response after just one PDT treatment. Only two cases were not free of disease. Three patients with palpebral carcinoma of the neck developed tumor recurrence, but only one required resection and PDT [64].

Ikeda et al. included 18 patients with squamous cell carcinoma and seven with epithelial dysplasia with oral hyperkeratosis in their study. A complete response was obtained in 96% of patients after PDT with Photofrin^®^. The disease-specific survival rate was 95.8%. Patients required medications to alleviate pain, which, along with swelling, appeared within 24 h after envenomation. The authors found no functional problems or aesthetic losses after the procedure [65].

Jerjes et al. applied PDT to patients with malignant oral diseases. The study group consisted of 147 patients treated with PDT using 5-ALA or 5,10,15,20-Tetrakis(3-hydroxyphenyl)chlorin (Temoporfin) The mean age was 53 years ± 8.9 years. There were 55 patients with homogeneous leukoplakia, 73 with heterogeneous leukoplakia, and 19 with erythroplakia. A complete response to treatment was obtained in 81% of the patients studied. In light of this, the authors suggest that 5-ALA and Tempoporfin PDT may become an effective alternative in the treatment of oral diseases [66].

Jerjes et al. included 38 patients with a mean age of 58 years with T1/T2 stage oral squamous cell carcinoma in their study. All patients were treated with Tempoporfin–PDT twice with an interval of 6–7 months after the first round. After the therapy, 26 patients showed a clinically completely normal oral cavity at the site of the primary tumor. The overall 5-year survival rate was 84.2%, and tumor recurrence was 15.8%. Recurrence very often involves ulceration of the buccal mucosa or the sinus area. Although three patients died due to the local or distant spread of the disease, they concluded that PDT may prove more effective than traditional interventions [67].

Wanitphakdeedecha et al. included 29 patients (26 women and 3 men) aged 21–39 years with facial acne of varying severity in their study. All subjects underwent PDT with facial division in which an active cream was used on one side and a carrier cream was used on the other side. The same treatment protocol was applied in both cases, twice a day for 10 weeks. After 2 weeks, the active cream group showed a faster rate of lesion reduction *p* = 0.010 for inflammatory acne and *p* = 0.001 for non-inflammatory acne. For non-inflammatory acne, the active cream worked better than the vehicle from the beginning to the end of therapy. One month after the fourth therapy, better efficacy of the active cream was also noted for inflammatory acne. The authors suggest the effective use of PDT combined with creams containing licochalcone A, L-carnitine, and decane diol in the treatment of acne [68].

Stoker et al. enrolled 21 patients with recurrent or residual nasopharyngeal cancer at a tumor depth of 10 mm or less. All received intravenous 0.15 mg/kg Tempoporfin 96 h before radiation. After 10 weeks, the tumor response to therapy was examined. This showed that 95% of patients had a complete response to the treatment, and two patients required repeat therapy, which was successful. The percentage of 2-year myocardial control was 75%, overall survival was 65%, and progression-free survival was 49%. At the end of the study period, 43% of patients were free of disease symptoms. The authors conclude that PDT may be an effective alternative in the treatment of nasopharyngeal carcinoma at a depth not exceeding 10 mm [69].

Jerjes et al. reported a study consisting of 33 patients with a mean age of 77.5 ± 8.3 years (90% Caucasian people) with advanced and/or recurrent cancer of the base of the tongue. All patients were treated with PDT using Tempoporfin 0.15 mg/kg intravenously 96 h before treatment. Six weeks after PDT, patients underwent radiological evaluation. In six patients there was no change in size, in seven patients there was a reduction of <25%, and in twelve patients, a reduction of <50%. In eight patients, there was a significant response to treatment and a 50–75% reduction in lesions. Six patients died due to the local or distant spread of the tumor. The authors emphasize that the patients included in the study were in a severe disease state and yet the results showed efficacy in controlling tumor progression and reducing tumor size [70].

Kübler et al. included 25 patients with flat epithelial lip cancer in their study. All patients had been administered 0.15 mg/kg Tempoporfin intravenously 4 days before exposure and participated in PDT. After 12 weeks, 96% of the patients showed a complete response to treatment. Recurrence of cancer was observed in two patients, one with metastases. Full functionality of the mouth was retained. The authors claim that Tempoporfin–PDT is an effective method of treating small primary lip tumors while maintaining the functionality of the lips and has a better aesthetic effect than traditional surgical treatment [71].

The efficacy and cost-effectiveness of PDT in actinic keratosis (AK) in the head and neck region that can lead to squamous cell carcinoma was also tested. The researchers concluded that 5-fluorouracil (5-FU) PDT was the most effective and cost-effective treatment for these patients, compared to imiquimod (IMQ) 5%, ingenol mebutate (IM) 0–015%, and methyl aminolevulinate photodynamic therapy (MAL-PDT) [72]. The efficacy of PDT in actinic keratosis was also explored by Buinauskaite et al. in a study in which the authors tested the efficacy of PDT using 5-aminolevulinic acid (ALA). In their conclusions, the researchers report that topical ALA-PDT with a light dose of 70 J/cm^2^ can be an effective treatment for mild to moderate lesions on the face and head, but adverse effects such as pigmentary changes and rosacea can occur [73].

## 4. Discussion

Cancer undoubtedly represents one of the greatest threats to life and health in the 21st century, thus constituting a major disease within the general population. There has been much progress in cancer prevention, but changing patients’ habits is one of the most difficult challenges for medical staff. According to GLOBOCAN—the Global Cancer Observatory—in 2020, the number of cancer cases will have risen to 19.3 million cases, and the number of cancer deaths is estimated to reach 10 million [74]. Cancer treatment can be divided into systemic treatment, which involves administering drugs systemically, and local treatment, which includes surgery and radiation therapy. Systemic treatment includes chemotherapies, hormonal therapies, and immunotherapies. The main side effects of radiation therapy are hair loss, general weakness, lack of appetite and associated weight loss, and diarrhea [75]. With chemotherapy, it is not uncommon for patients to complain of balance problems, sensory disturbances, or vomiting [72,73,76]. It is important to remember that in addition to the side effects of traditional treatment, which are acute, the side effects can be distant and appear even several years after the end of therapy. Therefore, cancer treatment is a challenge for the interdisciplinary treatment team, but most importantly for the patients themselves. The side effects of most traditional therapies, including chemo-radiotherapy and surgical methods, which compound poor mental state, psychological burden, prolong morbidity, and cause loss of function, can prove to be a greater burden on the patient than the diagnosis itself. Therefore, treatments are still being sought that minimize side effects, are effective as possible, and target the tumor mass.

### Limitations and Future Directions

PDT is used to treat various types of cancer. Despite the high efficiency of this treatment and its growing popularity, the limited depth of tissue penetration by light is becoming a serious problem. A milestone in solving this problem was the introduction of the concept of energy converters for converting the energy deposited by X-rays or γ-rays into luminescence in the optical range. Currently, a number of methods are being tested to support PDT and increase light penetration as well as the precision of PS delivery. Secchi et al. in their project, analyzed nano-sylators supporting PDT. The designed nano-sylators made it possible to control the efficiency of the non-radiative energy transfer process between the building blocks of a multicomponent system, i.e., a dense, flickering nanoparticle responsible for local interaction with ionizing radiation, consequently inducing ROS. In the future, purpose-designed nano-sylators will increase the effectiveness of the interaction of ionizing radiation with the PS, leading to a breakthrough increase in the effect of radiotherapy even at low doses [77]. Another example of advances supporting PDT is the development of organic-inorganic hybrids—so-called nanomaterials. Nanomaterials developed to date have a number of interesting physicochemical and biological properties that are highly valued in oncology applications. Hybrid nanosystems analyzed to date have favorable biointerfacial properties alongside unchanged physical and chemical properties. Examples of developed nanosystems include 2D/3D nanostructures with pharmaceutical and biological effects.

Nanostructures developed to date are multifunctional carriers for many elements. However, research on the analysis of the process of linking and correlation of structure and function between the carrier and the transported compound (drug, PS) is still in progress. Over the last 5 years, there have been many literature reports analyzing the molecular mechanism of the developed nanostructures and their effectiveness in biomedical applications. The results give hope that the developed nanostructures will enable the use of PDT in deeper organs, increasing the penetration of the PS and light [78]. Another scheme under development, which aims to improve penetration, and thus, increase the effectiveness of PDT, is radiosensitizers. Radiosensitizers developed to date have the ability to stop ionizing radiation and increase the locally delivered therapeutic dose. In turn, hybrid nanoparticles, composed of scintillators coupled with organic molecules, are a suitable tool in X-ray-activated PDT. Examples of nanoparticles studied to date include ZnO, HfO_2_, and Ta_2_O_5_ nanoparticles. Heavy metal nanoparticles or hybrids are one of the key prospects in the context of oncology therapy. The use of nanomedicine in the combination of radiotherapy and PDT is an important milestone in the development of oncology. The latest research studies focus on the development of hybrid systems and their application in diagnostics and therapy [79]. Similar observations were made by the research team of Secchi et al. In their review, they presented the principles of radiotherapy assisted by PDT, together with the mechanism and functioning of the supporting systems. The development of effective measures and strategies in PDT-assisted radiotherapy is a key step in the entire field of oncology therapy. The universality of an effective system is extremely important in the context of the treatment of internal organs to which access is difficult [80]. In turn, Shrestha et al. [81] proposed X-ray-induced PDT with copper-cysteamine nanoparticles in mouse tumors. The presented copper-cysteamine nanoparticles are one of the newest types of PS. One of their main advantages is the fact that they can generate cytotoxic ROS immediately after activation with X-rays. The designed model is a response to the still not fully resolved problem of limited light penetration in internal organs. The project used mouse breast cancer cells of the BALB/cRos strain (CRL-2116) and a mouse animal model BALB/c aged 3–4 weeks (both females and males). In total, six treatment groups with different sets of nanoparticles/radiation presence were analyzed. The tumor was ∼4–8 mm in size. Copper cysteamine nanoparticles (+ along with a low-pH insertion peptide) reduced tumor size in both sexes compared to mice treated with radiation alone (no nanoparticles). The authors proved that Cu-Cy nanoparticles conjugated to a low-pH insertion peptide can reduce tumor size in combination with radiotherapy in mice. The developed model of nanoparticles produced a better therapeutic effect than other methods [73].

## 5. Conclusions

PDT is gaining more and more supporters due to its selectivity and precision. Knowledge of the components of PDT is expanding and new properties are being discovered. When undertaking PDT, it is important to monitor oxygen concentration and light dose during therapy. There is still much debate over PS doses and radiation intensity. Many variables must be taken into account when choosing therapy components. When starting the selection, we consider the patient’s pain, the extent and size of the lesions, and the location. All of the cited studies have reached the common conclusion that PDT has the potential to reduce side effects and morbidity in many diseases, but research on the methodology for executing PDT in specific entities should continue.

## Figures and Tables

**Table 1:** Characteristics of selected photosensitizers.

Type of Photosensitizer	Activation Wavelength	Characteristics
Hematoporphyrin derivatives	620–650 nm	Absorb the light wave effectively at the certain wavelength
Phenothiazine (including toluidine blue and methylene blue)	620–700 nm	Have appropriate energy at the triplet state to provide sufficient energy at the transfer to the ground state
Cyanine	600–805 nm	Possess appropriate quantum yield
Phytotherapeutic agents	550–700 nm	Possess long lifetime at the triplet state
Phthalocyanine	660–700 nm	Have appropriate and high photostability

**Table 2:** Characteristics of second-generation photosensitizers.

Photosensitizer	Wavelength [nm]	Application
Ameluz^®^ (Biofrontera, Inc., Wakefield, MA, USA)/Levulan^®^	635	Mild to moderate actinic keratosis
Metvix^®^/Metvixia^®^	570–670	Non-hyperkeratotic actinic keratosis and basal cell carcinoma
Foscan^®^	652	Advanced head and neck cancer
Laserphyrin^®^ (Meiji Seika Pharma, Tokyo, Japan)	664	Early centrally located lung cancer
Visudyne^®^ (Novartis, Basel, Switzerland)	690	Age-related macular degeneration
Redaporfin^®^ (Luzitin SA, Coimbra, Portugal)	749	Biliary tract cancer
Fotolon	665	Nasopharyngeal, sarcoma
Radachlorin	662	skin cancer
Photochlor	664	Head and neck cancer
Tookad^®^	762	Prostate cancer
Antrin	732	Coronary artery disease
Photrex	664	AMD
Talaporfin	664	Colorectal neoplasms, Liver metastasis

## Data Availability

All data have been included.
